# Supplementary material for: The Repellent Capacity against Sitophilus zeamais (Coleoptera: Curculionidae) and In Vitro Inhibition of the Acetylcholinesterase Enzyme of 11 Essential Oils from Six Plants of the Caribbean Region of Colombia
Source: Molecules. 2024 Apr 12;29(8):1753. doi: 10.3390/molecules29081753 (PMC11051817; doi:10.3390/molecules29081753)
Supplement: Supplementary file 1 [file molecules-29-01753-s001.zip › molecules-2691346-supplementary.pdf]

Supplementary Material

The Repellent Capacity against *Sitophilus zeamais* (Coleoptera: Curculionidae) and In Vitro Inhibition of the Acetylcholinesterase Enzyme of 11 Essential Oils from Six Plants of the Caribbean Region of Colombia

Amner Muñoz-Acevedo [1,*], María C. González [1], Jesús E. Alonso [2] and Karen C. Flórez [2]

[1] Department of Chemistry and Biology; Universidad del Norte, Puerto Colombia, Colombia; amnerm@uninorte.edu.co, mgonzalesd@uninorte.edu.co

[2] Department of Mathematics and Statistics; Universidad del Norte, Puerto Colombia, Colombia; jcabrera@uninorte.edu.co, lozanok@uninorte.edu.co

* Correspondence: amnerm@uninorte.edu.co; Tel.: +57 605 3509509 ext. 3046

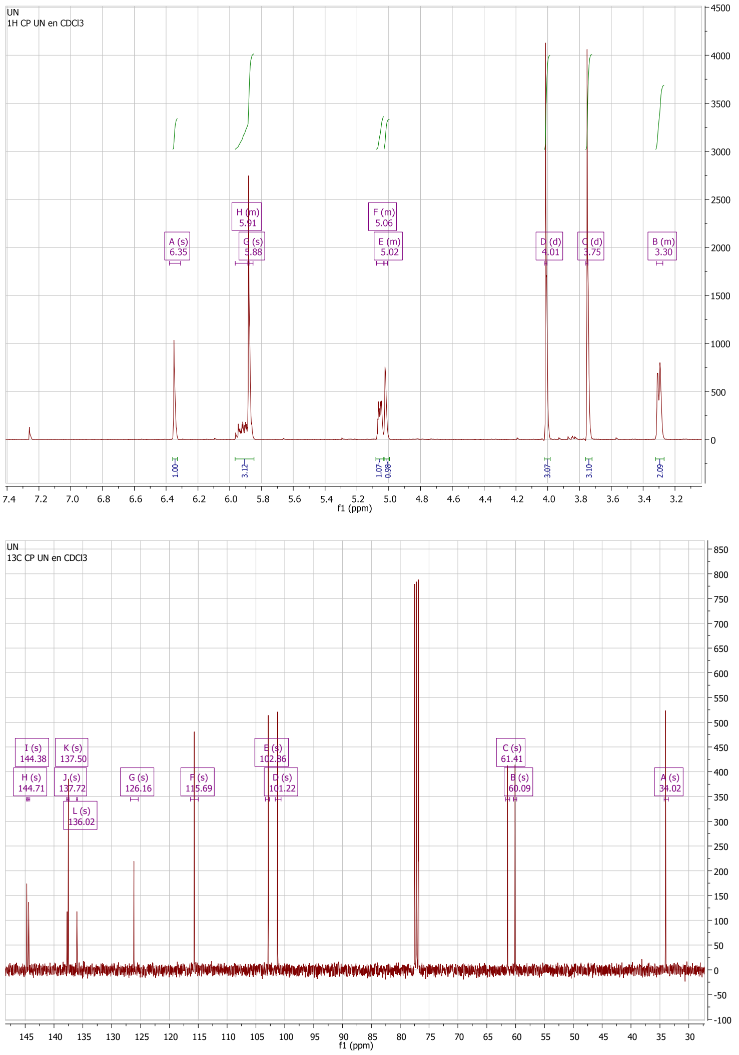

Figure S1. ^{1}H-/^{13}C-NMR spectra of the EO from *P. holtonii* (1b*) leaves.

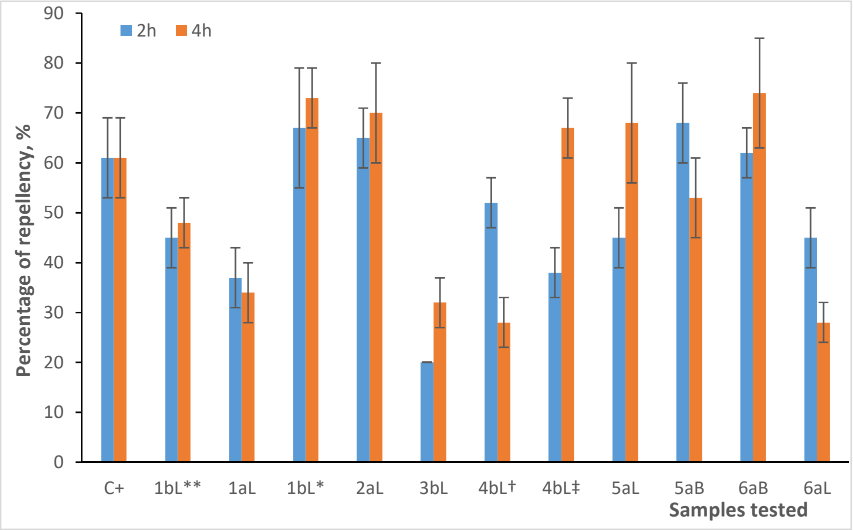

Figure S2. Repellent effect (at 2 h and 4 h) of 11 EOs and chlorpyrifos (C$^+$) against *S. zeamais*.

Two-way fixed-effect model (S1) related to ANOVA with two variables (exposure time and EO type)

$$Y_{ijk} = \mu + \alpha_i + \tau_j + (\alpha\tau)_{ij} + \varepsilon_{ijk}, \quad \text{(S1)}$$

where $i = 1,2,\ldots,12$; $j = 1,2$; $k = 1,2,\cdots, n_{ij}$; Y_{ijk} is the degree of repellency observed, α_i is the effect for the *i-th* treatment (EO type); τ_j is the effect for the *j-th* level of the exposure time factor; $(\alpha\tau)_{ij}$ represents the interaction between the two factors; and, ε_{ijk} corresponds to the random error associated with each response which fits a normal distribution (with zero as an average value and a constant standard deviation).

Table S1. Two-way ANOVA for the degree of repellency data.

Source of variation	SS	DF	MS	F value	Sign.
EO type	20674.436	11	1879.494	33.349	0.000
Time	182.932	1	182.932	3.246	0.075
Interaction	5907.309	11	537.028	9.529	0.000
Error	5804.861	103	56.358		
Total	399800.000	127			

SS—sums of square, DF—degrees of freedom, MS—mean square, [a]R^2: 0.827 (R^2_{adj}: 0.788).

One-way ANOVA combined with straight line regression (for a complete interaction) model (S2).

$$Y_{ij} = \mu + \alpha_i + \beta_i t_{ij} + \varepsilon_{ij}, \quad \text{(S2)}$$

where $i=1,2,\ldots,12$; $j=1,2,\ldots,n_i$; α_i is the effect of the *i-th* treatment (type of EO); t is the exposure time considered as a quantitative variable among 2–4 h, β_i is the slope which can depend on the level i of the first factor; and, ε_{ij} corresponds to the error associated with each response which is adjusted to a normal distribution.

Table S2. One-way ANOVA combined with simple linear regression for the degree of repellency data.

Source of variation	SS	DF	MS	F value	Sign.
EO type	5852.636	11	532.058	9.441	0.000
Exposure time (EO type)	6000.335	12	500.028	8.872	0.000
Error	5804.861	103	56.358		
Total	399800.000	127			

SS—sums of square, DF—degrees of freedom, MS—mean square, [a]R^2: 0.827 (R^2_{adj}: 0.788).

Table S3. Significant cases (EO type) in the ANOVA model (2) with exposure time as a covariate.

Parameter	B	SE	t	Sig.	Parameter	B	SE	t	Sig.
Interception	60.139	4.150	14.492	0.000					
α_1	−17.639	9.363	−1.884	0.062	β_1	1.250	2.654	0.471	0.639
α_2	−20.806	10.180	−2.044	0.044	β_2	−1.333	2.741	−0.486	0.628
α_3	−0.139	10.543	−0.013	0.990	β_3	3.333	3.065	1.088	0.279
α_4	−0.139	9.211	−0.015	0.988	β_4	2.500	2.518	0.993	0.323
α_5	−52.639	9.363	−5.622	0.000	β_5	6.250	2.654	2.355	0.020
α_6	17.361	9.363	1.854	0.067	β_6	−12.500	2.654	−4.710	0.000
α_7	−51.806	9.611	−5.390	0.000	β_7	14.583	2.867	5.087	0.000
α_8	−38.472	9.109	−4.224	0.000	β_8	11.667	2.423	4.815	0.000
α_9	22.528	8.468	2.660	0.009	β_9	−7.333	2.273	−3.226	0.002
α_{10}	−9.139	9.211	−0.992	0.323	β_{10}	5.750	2.518	2.284	0.024
α_{11}	1.861	9.211	0.202	0.840	β_{11}	−8.500	2.518	−3.376	0.001
α_{12}	0				β_{12}	0.243	1.290	0.188	0.851

B—Estimated value of parameter, SE—Standard error, t—t value.

Table S4**.** Inhibitory effects (IC_{50} and %I (at 1 μg/mL)) on AChE of the 11 EOs and the control substance (chlorpyrifos).

**Code**	**Sample evaluated**	**Inhibitory effects on AChE**[Ŧ]	
		**IC_{50} (μg/mL)**	**I% (at 1 μg/mL)**
C[+]	Chlorpyrifos	0.59 ± 0.04	59 ± 3
1bL**	*P. holtonii**	32 ± 2	NR
1aL	*P. holtonii*	38 ± 2	NR
1bL*	*P. holtonii*	28 ± 2	NR
2aL	*Pep. pellucida*	14 ± 1	NR
3bL	*P. haugtii*	40 ± 3	NR
4bL[†]	*P. reticulatum*	21 ± 1	NR
4bL[‡]	*P. reticulatum*	25 ± 2	NR
5aL	*B. graveolens*	16 ± 1	NR
5aB	*B. graveolens*	18 ± 1	NR
6aB	*B. simaruba*	7.7 ± 0.9	NR
6aL	*B. simaruba*	4.6 ± 0.3	10.4 ± 0.5

L—Leaves, B—Bark, a—Atlántico, b—Sucre, * Location I, ** Location II, [†] Fresh, [‡] Dried, [Ŧ] Values reported as $\bar{X}$ ± s according to the replicates. Codes for plants are numbers: different types of species (1—*P. holtonii,* 2—*Pep. pellucida,* 3—*P. haugtii*, 4—*P. reticulatum*, 5—*B. graveolens,* 6—*B. simaruba*).
